# Reconciling Paid Work and Informal Caregiving Among Older Adults: Implications for Work Interference With Family

**DOI:** 10.1093/geroni/igad115

**Published:** 2023-10-06

**Authors:** Shanika Yoshini Koreshi, Fiona Alpass

**Affiliations:** School of Psychology, Massey University, Manawatū, New Zealand; School of Psychology, Massey University, Manawatū, New Zealand

**Keywords:** Employment, Informal caregiving, Older adults

## Abstract

**Background and Objectives:**

Many older adults who combine paid work and informal caregiving responsibilities are often in work arrangements that differ from their preference. There is reason to believe that such work status incongruence may lead to work interference with family (WIF). In response, many governments have policies that support flexible work arrangements (FWA) to help individuals manage work and family commitments. This paper examines whether work status preferences contribute to WIF for older adults who combine work and care and whether the use of flexible work arrangements moderates this relationship.

**Research Design and Methods:**

The study included 610 informal caregivers (aged 55–70 years) in paid employment who participated in either the 2018 or 2020 waves of the New Zealand Health, Work and Retirement study. Using a simple moderation analysis with demographic controls, the effects of work status preferences and FWAs on WIF were estimated.

**Results:**

After controlling for confounds, significant main effects were found for both work status preferences and flexible work arrangements on WIF. The moderation analysis revealed that involuntary part-timers using flexible time-off arrangements reported lower levels of WIF. However, other types of flexible work arrangements did not moderate the relationship between work status preferences and WIF.

**Discussion and Implications:**

These findings suggest that work status preferences can contribute to WIF, but not all types of flexible work arrangements alleviate it. Policy initiatives designed to reduce WIF for those combining work and care should consider accounting for differences in the work status preferences of older workers.

**Translational Significance:** Combining paid work and informal caregiving among older adults is understudied. The growing aging population and the need for informal caregivers highlight the need for ongoing research and discussions. Therefore, it is imperative to re-evaluate existing support systems and policies. This paper introduces work status preferences as a nuanced variable to study work–family conflict among older adult caregivers. The paper also proposes the importance of employers and policymakers recognizing the limitations of current flexible work arrangements and designing arrangements that align with the unique work challenges of older workers juggling paid work and caregiving responsibilities.

The rapid growth of an aging population, care demands, and healthcare costs have contributed to a shift from formal to informal caregiving (Lindt et al., 2020). To keep up with increasing care demands, family members are increasingly combining paid employment with caregiving responsibilities (Boumans & Dorant, 2021). An increasing aging population and the consequent rise of older workers in the workforce have brought attention to the challenges they face in reconciling paid work with informal caregiving responsibilities. Balancing work and family obligations is a complex task, particularly for older adults who often encounter unique circumstances that can impact their ability to manage both roles effectively. Older adults, often confront age-related health issues increased caregiving demands, workplace barriers, and limited support systems, which can significantly impact their ability to balance work and family obligations (Cebola et al 2021; Clancy et al 2020; Fried et al, 2001). Currently, there is a dearth of research that investigates the context of work, and in particular work–family conflict, and caregiving among older workers. Existing studies have predominantly focused on employees below the age of 55 years (Bainbridge, Palm & Fong, 2021; Tement & Korunka, 2013), neglecting the distinct circumstances and considerations faced by older workers. This study aims to bridge the existing research gap and provide actionable insights for organizations and policymakers to develop age-inclusive policies and support workplace environments that cater to the needs of older employees.

There is evidence that older adults who take up caregiving have work preferences that do not match their actual work status (Koreshi & Alpass, 2022) and that those in involuntary part-time work (preference for full-time work) are more likely to take up care. When preferences do not match actual work status/work hours individuals may experience work status incongruence (Holtom et al., 2002). There is reason to believe that work status incongruence may lead to work interference with family (WIF). Studies have shown that individuals who experience a state of fit between desired and actual work hours have less conflict between work and life (Lu, 2011), although work hour mismatches can constrain the ability to reconcile family and work responsibilities (Jacobs & Gerson, 2005). For instance, Brauner et al. (2020) found that satisfaction with work–life balance decreased if employees worked more than their preferred hours, and misfits in preferred work schedules led to work-to-family conflict (Piszczek et al., 2021). Thus, for some working carers, WIF may arise not only from balancing work and family responsibilities (Lee et al., 2010) but also from not having their work status preferences met.

Flexible work arrangements (FWAs) have been proposed as a way to help individuals manage work and family care commitments (Shockley & Allen, 2007) and have been a focus of government policy in recent years (MSD, 2009; Fair Work Ombudsman, 2018). However, empirical studies have produced inconsistent results when examining the relationships between FWAs and work interference with family (Allen et al., 2013) with small effect sizes and associations dependent on the nature of the work–family conflict and the type of flexible work arrangements. Work status preferences may also play a role in this relationship. For example, workers who are in involuntary full-time work (prefer reduced hours) may benefit, in terms of WIF, from enhanced control over their start and finish times, or by having the option of compressing their work week. Similarly, for those who are in involuntary part-time work (prefer more hours), FWAs may offer opportunities for scheduling non-work activities such as caregiving responsibilities in order to reduce WIF.

This study aims to understand the role of work status preferences and flexible work arrangements in WIF among older workers who combine paid work and informal caregiving. In the present study, an individual was classified as an informal caregiver if they provided unpaid care for someone with either a long-term illness, frailty, or disability. Care recipients included spouses, parents, children, parents-in laws, and other family and friends. Specifically, the study addresses the following research questions: (a) Are work status preferences a source of WIF for older adults who combine paid work and informal caregiving? and (2) Do flexible work arrangements moderate the relationship between work status preferences and WIF? Given that work–family conflict is considered a public health concern (Chandler, 2021), understanding the role of work status preferences and flexible work arrangements in mitigating WIF can help inform policies and interventions aimed at improving the well-being of working carers.

## Literature Review

### Work–Family Conflict

*Work–family conflict* is a common experience for many individuals, resulting from the inter-role the conflict of between the energy, time and behavioral demands of work and family roles (Greenhaus & Beutell, 1985), making it difficult to complete or meet demands in both roles. Conceptually, conflict between work and family is bi-directional. The two established directions of work–family conflict are family interference with work (FIW) and work interference with family (WIF; Allen et al., 2013). WIF and FIW are two related but distinct constructs, justifying separate examinations (Allen et al., 2013, Mesmer-Magnus & Viswesvaran, 2006). Extensive research consistently shows that WIF occurs more frequently than FIW (Frone, 2003). This supports the domain specificity hypothesis, indicating that work-related factors are closely linked to WIF rather than FIW (Frone et al., 1997). Thus, the present study will focus on studying work interference with family (WIF).

Work interference with family is associated with a range of negative health outcomes including stress, anxiety, and depression (Borgmann et al., 2019) and is also linked to low levels of job satisfaction (Bruck et al., 2002). In particular, informal caregivers who combine work and care may experience greater work–family conflict when satisfying the demands of each role becomes more challenging (Kayaalp et al., 2021; Lee et al, 2010).To reduce work interference with family, researchers have explored various organizational practices, including flexible work arrangements (Glass & Estes, 1997) and work–family policies (Voydanoff, 2005).

Job demands, such as long work hours and high levels of job responsibilities, have been identified as important determinants of work interference with family (Allen et al., 2013). However, the relationship between employment status (i.e., full-time vs part-time) and work interference with family is complex and inconsistent. Some studies suggest that working part-time may reduce work interference with family by allowing individuals to balance work and family responsibilities (Bianchi & Milkie, 2010). Other studies have reported no effect on part-time employment (Warren, 2004).

The inconsistencies in the literature may be due in part to differences in employee preferences for working full-time or part-time. The current study distinguishes between regular part-time and involuntary part-time workers who want to work full-time, as well as regular full-time workers and workers who want to work part-time. By taking into account employee preferences, this study aims to provide a more nuanced understanding of the relationship between work hours and WIF.

### Work Status Preference

Organizational behavior literature identifies ability and preferences as the basic dimensions of fit (Kalleberg, 2008). Two main kinds of such temporal mismatches of preference are identified as underworking (working less than one prefers) and overworking (working more than one prefers; Kalleberg, 2008). An individual who is working fewer hours than they desire may experience a state of misfit, which is likely to increase stress (Lu, 2011). Lu (2011) emphasizes that to enhance personal and social welfare we need to understand an individual’s motivation and/or preference to do more or less work. This becomes more critical among older workers who combine paid work and care, as they experience more barriers in remaining or returning to work (Muller & Volkov, 2009).

#### Why study mismatches/preferences?

Studying mismatches or discrepancies between employees’ preferred and actual work status is important for several reasons. First, failure to provide employees with their preferred work hours may negatively impact both organizational performance and employee job satisfaction (Van Emmerik & Sanders, 2005). Employees who work fewer hours than they prefer may experience lower levels of organizational citizenship and meaningful work (Kim & Allan, 2020), while those who work more hours than they prefer may report higher levels of work interference with family (Reynolds, 2005). All of these outcomes can impede the effectiveness of a workforce.

Second, mismatches in work hours can make it more challenging for individuals to fulfill caregiving responsibilities and provide adequate financial support for their families (Reynolds & Aletraris, 2007). Although some individuals may prefer to work fewer hours, the labor market may offer limited options (Clarkberg & Moen, 2001). Conversely, if individuals work fewer hours than they prefer, they may experience financial difficulties, which can in turn affect their family’s well-being.

It is generally assumed that people prefer to work fewer hours as they approach retirement age (Van Solinge & Henkens, 2014). However, research suggests that some older adults may prefer to work more hours (Koreshi & Alpass, 2022; Silver et al., 2019). A range of individual, societal, and work-related factors such as gender, financial status, health status, and supportive work environments can contribute to workers’ preferences regarding hours worked (Bell & Rutherford, 2013). Furthermore, not all older workers with caregiving obligations necessarily wish to work less; some may prefer more work to increase their income in order to hire professional help to assist them with caregiving responsibilities (Kunze et al., 2011). Therefore, the assumption that older workers prefer less work as they approach retirement age may not apply to older adults who combine paid work and caregiving responsibilities. Furthermore, if older workers drop out of the labor force prematurely due to caregiving demands, it may lead to a loss of skilled workers and reduce the overall productivity of the workforce. Understanding their work preferences can inform policies that promote work–life balance and workforce participation.

### Flexible Work Arrangements

Flexible work arrangements (FWA) have been increasingly recommended as a means to help individuals effectively balance their work and family commitments. Older adults who simultaneously manage care and work responsibilities commonly utilize FWA options such as flexible schedules, time off, and flexible work hours (Koreshi & Alpass, 2023). However, empirical studies have reported mixed findings when examining the relationships between FWAs and work–family conflict (Allen et al., 2013). Effect sizes associated with various types of FWA and work–family conflict have ranged from −0.01 (Mesmer-Magnus & Viswesvaran, 2006) to −0.30 (Byron, 2005). It is important to note that prior research has not distinguished between the use and availability of flexibility when attempting to explain the variation in the relationship between flexibility work–family conflict (Allen et al., 2013).

Having access to FWAs can promote positive attitudes toward one’s organization (Batt & Valcour, 2003) and enhance perceptions of psychological control (Kossek et al., 2006). According to the social-exchange theory, an employee with access to flexible work arrangements is likely to reciprocate positive feelings towards their job. However, the availability of flexible work arrangements may not always align with the flexibility an employee needs. Moreover, some employees may not use FWA due to perceived or actual negative effects on career advancement and financial consequences (Fursman & Zodgekar, 2009).

Alternatively, Kossek et al. (2006) argue that using flexible work arrangements will increase psychological control while providing employees with tangible strategies to manage role boundaries. The use of flexibility enables employees to proactively structure and manage responsibilities that originate from work and family. Thus, it is argued that FWA use is more likely to buffer against work interference with family than access to FWA alone (Allen et al., 2013). Studying utilization allows us to capture the lived experiences of individuals who actively incorporate FWAs into their work and caregiving routines. Therefore, the present study will examine the use of flexible work arrangements among older workers who reconcile paid work and caregiving responsibilities, with the aim of better understanding how FWAs can help mitigate work interference with family.

Different types of FWAs are commonly offered by organizations, and employees may have preferences for or use specific types of arrangements based on their varying needs and priorities. The present study investigates the use of four distinctive types of flexible work arrangements (flexibility in the number of hours worked, flexible place, time off, and flexible schedules). By examining the use of different types of FWAs, our study aims to capture the diversity of work arrangements and understand how the alignment or misalignment between work status preferences and specific types of FWAs may influence work interference with family. This approach acknowledges the multidimensionality of work flexibility and its implications for work–life balance outcomes.

### Theoretical Framework and Present Study

The phenomenon of “work mismatches” can be explained through the lens of the demands–control model of strain (Karasek, 1979; Karasek and Theorell, 1990), a widely used theoretical framework in the study of work-related stress. According to this model, individuals experiencing high work demands and inadequate resources are at the greatest risk of experiencing strain. In order to examine work interference with family, we adopt a demands-and-resources approach (Voydanoff, 2005) inspired by Karasek’s model. This approach divides work-related factors into two main categories: demands and resources. Work demands pertain to the requirements of the work role, which often involve physical or mental exertion and are associated with time or energy costs. Examples of work demands include working more or fewer hours than desired. Work resources, on the other hand, are assets that can be used to manage demands, such as flexible work arrangements (Voydanoff, 2005).

In the context of work, preferences can represent an individual’s desired conditions or outcomes related to their work. Discrepancy theory suggests that positive work outcomes arise when there are fewer mismatches between an employee’s desires and the job requirements, they are expected to fulfill (Locke, 1969). When workers’ preferences are not met, it can create a sense of discrepancy or misalignment between their desired and actual work situation. This discrepancy can lead to additional psychological or emotional strain, which can be seen as a form of demand (Rice et al., 1990). This misalignment between preferences and actual work conditions can create additional stress and dissatisfaction among workers and may even increase their perceived strain as they try to cope with managing those work demands and providing care.

Similar to the work of Steiber (2009), we differentiate between two types of work demands: time-based demands and strain-based demands. Time-based demands reflect the notion that time is a limited resource, and hence time spent on work reduces the amount of time available for family-related activities. For instance, an involuntary full-time work schedule could be a time-based demand. Strain-based demands, on the other hand, may produce negative experiences, such as feelings of job insecurity that can result from working fewer hours than desired (involuntary part-time work). This situation may threaten the economic well-being and stability of the family for some individuals. There is some evidence to suggest that using work resources such as flexible work arrangements may potentially alleviate the negative impact of work demands on well-being (Ray & Pana-Cryan, 2021). For instance, employees with a preference for fewer working hours may experience higher levels of work interference with family when they are unable to meet their preferred work status. However, if they have access to FWAs such as paid time off or flexible scheduling, they may be able to adjust their work arrangements to better accommodate their family responsibilities. This, in turn, can reduce work interference with family, as they have greater control over managing their time and balancing their work and personal life.

Involuntary part-time work can be a source of financial strain and job insecurity (Kretsos & Livanos, 2016), and employees who desire more working hours but are unable to secure them may experience a sense of unmet demand, potentially leading to work–family conflicts. Flexible work arrangements (FWAs) can play a role in alleviating work demands and enhancing work–life balance. For individuals seeking to increase their work hours, FWAs like “input into overtime hours” or “the number of hours worked” could prove beneficial. By allowing employees to express their willingness to work additional hours or having a say in scheduling, they may have better opportunities to secure extra work hours or build a stronger relationship with the organization, increasing their potential for more permanent hour adjustments.

Drawing on Karasek’s demand–control model of work-related strain and the demands-and-resources approach (Voydanoff, 2005), we propose the following hypotheses:

H1: Older adults in involuntary part-time and involuntary full-time work will report higher levels of WIF than older workers in voluntary part-time and voluntary full-time work.

H2: Flexible work arrangements will be negatively associated with WIF.

The moderating role of FWAs in the relationship between work status preferences and work interference with family is based on the premise that these arrangements offer employees the flexibility to align their work commitments with their personal and family responsibilities. By enabling individuals to adapt their work arrangements to suit their preferences, FWAs can help alleviate the negative effects of unmet work status preferences on work interference with family.

H3: The relationship between work status preferences and WIF will be moderated by flexible work arrangements.

In sum, we respond to the call of previous authors (Clancy et al., 2020; Kossek et al., 2011; Moussa, 2019) to explore how older workers reconcile the responsibilities of juggling paid work and care. We address the limitations of previous research in the following ways: (a) We examine the use of flexible work arrangements as opposed to perceived access. (b) We account for individual work preferences when examining antecedents of WIF. (c) We examine if flexible work arrangements moderates the relationship between work preferences and WIF.

## Method

### Design

The proposed research will utilize existing secondary data gathered from the Health, Work and Retirement (HWR) longitudinal study. The HWR study is a population-based study that aims to identify the health, economic, and social factors underpinning aging in Aotearoa New Zealand. Participant cohorts in the HWR study have been drawn from a large random sample of people aged 55 years and over who are listed on the New Zealand electoral roll. Since its launch in 2006, participants in the HWR study have been re-approached every two years to complete the survey. Data from the 2018 and 2020 biennial surveys were used in the present research to optimize the sample size.

### Sample

Participants who responded to Wave 7 (2018) or Wave 8 (2020) were included in the present study. A total of *N* = 3,965 returned completed surveys in 2018 and *N* = 4,344 in 2020. Overall, *N* = 5042 adults aged 55+ responded to one or more surveys in 2018 and 2020. For the current analyses, participants who were in paid employment, aged 55–70 years, and who identified as caregivers were included. Therefore, we excluded participants (a) aged over 70 years due to overall levels of workforce participation in this age group, (b) who did not provide data on WIF, (c) who had missing information about their current and preferred employment status, and (d) those who identified as noncaregivers. This resulted in an analytic sample of 610 participants. The study was conducted with approval from the Massey University Human Ethics Committee: Southern B Application 09/70.

### Materials

#### Caregiving status

To enable representation of a range of low- to high-intensity caregiving conditions, participants were classified as caregivers if they reported that they had provided practical assistance to someone with a long-term illness, disability, or frailty for, at least, 3 hr a week in the past 12 months.

#### Work interference with family

WIF was measured using a single item “My job makes it difficult to be the kind of spouse or parent I’d like to be.” This item assessed the extent of the inter-role conflict between work and family roles. The responses are obtained using a 5-point Likert-type scale where 1 equal strongly disagree, and 5 equals strongly agree.

#### Work status preferences

A mismatch between a participant’s preferred and current work status was used to categorize work status incongruence/preference. Participants who worked part-time but preferred to work more were categorized as (1 = involuntary part-timers), participants who worked full-time and preferred to work less were categorized as (2 = involuntary full-timers), participants who identified part-time work as their preferred and current work status were categorized as (3 = voluntary part-timers), and participants who were in full-time work and preferred the same were categorized as (4 = voluntary full-timers).

#### Flexible work arrangements

Participants were asked to review 17 flexible work arrangement policies (adapted from Rudolph & Baltes, 2017), generally applicable to a variety of jobs, and indicate whether they had used them or not. These work arrangements were then categorized into four distinct types based on the typology outlined in Pitt-Catsouphes et al. (2009): Flexibility in number of work hours (5 items), flexible schedule (5 items), flexible place (2 items), and options for time off (5 items). For each of the four flexible work arrangement types, participants’ responses were individually scored, with a value of 1 assigned for use and a value of 0 for nonuse. Thus, the scores for the four categories of FWAs were maintained separately, reflecting the level of use in each type. A higher score within each specific flexible work arrangement category indicated a greater extent of use for that particular type of arrangement.

This approach allowed for a detailed examination of the participants’ use of each flexible work arrangement type independently, providing insights into their preferences and patterns of adoption for each category of FWAs.

#### Covariates

Demographic indices were included as covariates. These included gender (male = 0, female = 1) and socioeconomic status (SES). Socioeconomic status was measured using the economic living standards index (Jensen et al., 2005). This 25-item scale measures participants’ financial and economic well-being. It is a nonmonetary indicator of SES in New Zealand that measures restrictions in social participation, restrictions in ownership of assets, economizing behavior, and self-reported standard of living. A total score can be derived by summing all the items with a range of 0–31. A higher score corresponds to a higher level of economic living standards.

### Statistical Analyses

Descriptive statistics for the study population are provided as frequencies and percentages for categorical variables and as means and standard deviations (*SD*) for flexible work arrangements and WIF scores. The bivariate associations of key study variables and covariates with WIF scores were assessed using independent *t-*tests and analysis of variance (ANOVA).

To investigate whether flexible work arrangements moderate the relationship between work status preferences and WIF a simple moderation analysis was performed using Model 1 PROCESS macro in SPSS version 27.

## Results

Table 1 demonstrates that the majority of the caregivers are females, married, and in nonprofessional jobs. More than half of the respondents reported having good economic living standards. The current work status and preferred work status were incongruent for approximately 25 % of caregivers.

**Table 1.** Descriptive Statistics for Sample Characteristics

Characteristics	*N*	%	M (*SD*)
Gender			
Female	208	34.2	
Male	401	65.7	
Missing	1	0.2	
Age	610		
Marital status			
Married	477	78.2	
Single	123	20.2	
Missing	10	1.6	
Education qualifications			
No qualifications	82	13.4	
Secondary school qualifications	133	21.8	
Post-secondary certificate, diploma, or trade diploma	209	34.7	
University degree	178	29.6	
Missing	8	1.3	
Occupation			
Professional	159	20.6	
Nonprofessional	422	72.6	
Missing	29	4.8	
Economic living standards			
Hardship	114	18.7	
Comfortable	118	19.3	
Good	349	57.2	
Missing	29	4.8	
Work status preference			
Involuntary part-time	33	5.4	
Involuntary full-time	124	20.3	
Voluntary part-time	195	32.0	
Voluntary full-time	258	42.3	
Missing	0	0.0	
Flexible work arrangements			
Flexible place	610		1.7 (0.55)
Flexible schedule	610		3.8 (1.23)
Time off	610		3.6 (1.2)
Flexibility in the number of work hours	610		4.0 (1.2)
Missing	0		
WIF	610		2.1 (1.3)
Missing	0		

*Notes*: WIF = work interference with family.

On average, participants were 62 years of age with 23.0% having passed the eligible age for an aged pension. The sample exhibits some diversity, comprising 65.7% of female workers and 62.6% in full-time employment, although 20.3 % of these participants preferred to work less. Just 29.6% held a tertiary qualification and 20.6% reported to be in professional jobs such as accountant, doctor, nurse, and teacher.

Table 2 shows the correlations among the four types of flexible work arrangements and WIF. Using flexible work schedules and time off arrangements were significantly associated with lower levels of WIF. There were no significant associations between the remaining two types of flexible work arrangements, namely, flexible place and flexibility in the number of hours worked, and WIF.

**Table 2.** Estimated Correlations Between the Four Types of FWAs and WIF.

Variables	Flexible place	Flexible schedule	Flex time off	Flex number of hours
WIF	0.01	−0.12*	−0.08*	0.04

*Notes*: FWA = flexible working arrangements; WIF = work interference with family.

* *p* < .05.

To test the hypothesis that using flexible work arrangements moderates the relationship between work status preferences and WIF, a series of moderation analyses were undertaken using HAYES Process Version 4 for each of the four FWA categories. To avoid potentially problematic high multicollinearity with the interaction term and the variables were centered (Aiken et al., 1991). Analyses for a flexible schedule, flexible place, and flexibility in a number of work hours showed no significant main effect or interaction between the key variables (see Table 4, Table 5 and Table 6). Time off was the only FWA category that showed significance (see Table 3).

**Table 3.** Hayes’ Process Macro Results for the Test of Hypotheses (Time Off Arrangement)

Variable	*B*	*t*	*p*	95% CI
Work status preferences (reference group VFT)				
IVP	0.21	0.87	.38	(–0.25, 0.69)
IVF	0.49	3.97	.000*	(0.22, 0.71)
VP	–0.45	–3.11	.001*	(–0.72, –0.15)
Time off	–0.54	–2.73	.005*	(–0.91, –0.15)
IVP × time off	0.57	2.49	.013*	(0.11, 1.01)
IVF × time off	0.03	0.26	.80	(–0.20, 0.28)
VP × time off	–0.07	–0.66	.51	(–0.27, 0.13)
Gender	–0.20	0.11	.07	(–0.41, 0.02)
ELSI	–0.27	0.06	.000*	(–0.38, –0.12)
Survey year	0.07	0.64	.52	(–0.15, 0.29)

*Notes*: ELSI = economic living standards index; IVF = involuntary full-time; IVP = involuntary part-time; VFT = voluntary full-time; VP = voluntary part-time

*p < .05.

**Table 4.** Hayes’ Process Macro Results for the Test Moderation (Flexibility in the Number of Hours Worked)

Variable	*B*	*t*	*p*	95% CI
Work status preferences (reference group VFT)				
IVP	0.02	0.03	.97	(–1.16, 1.13)
IVF	0.66	1.24	.22	(1.71, 0.39)
VP	–0.09	–0.16	.87	(–1.17, –0.99)
Flexibility in the number of hours	–0.07	–0.57	.57	(–0.31, –0.17)
IVP × flexibility in the number of hours	0.11	0.80	.42	(–0.16, 0.38)
IVF × flexibility in the number of hours	0.09	0.73	.46	(–0.15, 0.34)
VP × flexibility in the number of hours	0.05	0.39	.70	(–0.20, –0.30)
Gender	–0.19	0.05	.0003*	(–0.30, –0.09)
ELSI	–0.05	0.004	.000*	(–0.06, –0.04)
Survey year	–0.05	0.05	.32	(–0.16, 0.05)

*Notes*: VFT = voluntary full-time; IVP = involuntary part-time; IVF = involuntary full-time; VP = voluntary part-time; ELSI = economic living standards Index.

* *p* < .05.

**Table 5.** Hayes’ Process Macro Results for the Test Moderation (Flexible Place)

Variable	*B*	*t*	*p*	95% CI
Work status preferences (reference group VFT)				
IVP	–0.02	–0.05	.96	(–0.88, 0.83)
IVF	–0.89	–2.16	.03*	(–1.69, –0.08)
VP	–0.38	–0.93	.35	(–1.17, 0.42)
Flexible place	–0.31	–1.47	.14	(–0.73, 0.10)
IVP × flexible place	0.27	1.12	.26	(−0.21, 0.75)
IVF × flexible place	0.35	1.54	.12	(−0.10, 0.80)
VP × flexible place	0.29	1.28	.20	(−0.15, −0.73)
Gender	−0.19	−3.65	.0003*	(−0.30, −0.09)
ELSI	−0.05	−11.3	.000*	(−0.06, −0.04)
Survey year	−0.06	−1.02	.31	(−0.16, 0.05)

*Notes:* ELSI = economic living standards index; IVF = involuntary full-time; IVP = involuntary part-time; VFT = voluntary full-time; VP = voluntary part-time.

* *p* < .05.

**Table 6.** Hayes’ Process Macro Results for the Test Moderation (Flexible Schedule)

Variable	*B*	*t*	*p*	95% CI
Work status preferences (reference group VFT)				
IVP	0.07	0.16	.88	(−0.88, 0.97)
IVF	−0.29	−0.68	.50	(−0.83, 0.97)
VP	0.09	0.22	.83	(−0.74, 0.93)
Flexible schedule	0.04	0.43	.67	(−0.15, 0.24)
IVP × flexible schedule	0.10	0.84	.40	(−0.13, 0.32)
IVF × flexible schedule	0.001	0.005	.10	(−0.21, 0.21)
VP × flexible schedule	0.004	0.03	.97	(−0.20, 0.21)
Gender	−0.19	−3.69	.0002*	(−0.30, −0.09)
ELSI	−0.05	−11.3	.000*	(−0.06, −0.04)
Survey year	−0.06	−1.04	.30	(−0.16, 0.05)

*Notes*: ELSI = economic living standards index; IVF = involuntary full-time; IVP = involuntary part-time; VFT = voluntary full-time; VP = voluntary part-time.

*p < .05.

### Interaction Effect of Time Off

Among the work status preferences, involuntary full-time workers (IVF) reported significantly higher WIF compared with the reference group (voluntary full-time workers). Voluntary part-time workers (VP) showed a significant negative association with WIF, indicating lower WIF levels than voluntary full-time workers.

**Figure 1.**
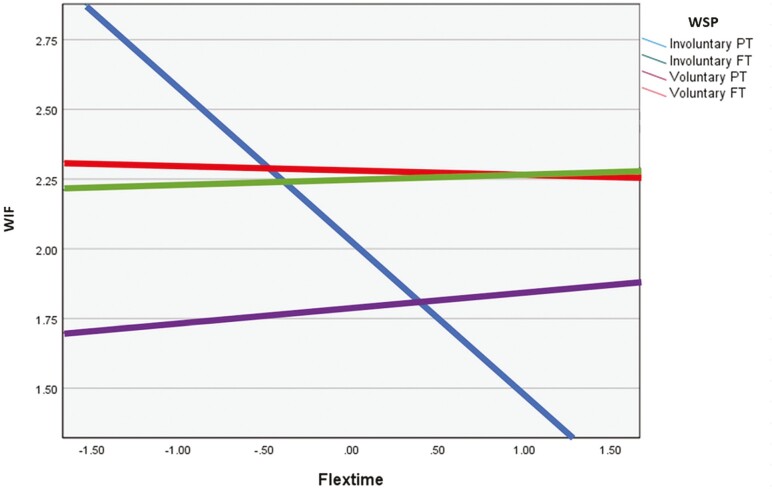
Interaction plot (time off arrangements).

The interaction effect between involuntary part-time and time off was significant, suggesting that the relationship between work status preferences and WIF is moderated by the use of time off arrangements for this group. It also indicates that the moderating role of flexible time-off arrangements on WIF is larger among involuntary part-timers than among voluntary full-timers. Examination of the interaction plot showed that as use of time off arrangements increased for involuntary part-time workers, WIF decreased (see Figure 1). The interaction effect between IVF and time off was not significant, neither was the interaction effect between VP and time off, indicating that the relationships between these two work preferences and WIF do not significantly vary based on the use of time off arrangements.

Gender and economic living standards index (ELSI) were also included as covariates in the analysis. Gender did not show a significant association with WIF, while ELSI was significantly related to WIF, indicating that higher ELSI scores were associated with lower WIF levels. The inclusion of the survey year (2018 and 2020) in the analyses aimed to examine and control for any potential impact of coronavirus disease (COVID-19) during 2020 on the use of flexible work arrangements and work interference with family. The survey year was not statistically significant, suggesting that COVID-19 did not have a significant impact on these variables in the current analysis.

## Discussion

The aim of the study was to explore the association between work status preferences and WIF among older adult caregivers and to examine whether flexible work arrangements moderated this relationship.

The first hypothesis received partial support. Specifically, participants who were employed involuntarily full-time reported higher levels of WIF than those in other groups, including voluntary part-timers, voluntary full-timers, and involuntary part-timers. Additionally, those in involuntary part-time work reported more WIF than voluntary part-time caregivers.

WIF arises from the challenges of balancing demands between work and family roles. Previous research has found that employees who work more hours than they prefer are likely to experience high levels of WIF (Reynolds, 2005). This is particularly relevant for caregivers who are overemployed, as they must navigate the demands of both caregiving and work roles simultaneously (Kayaalp et al., 2021; Lee et al., 2010).

Involuntary part-time workers, who would prefer to work more hours, also experienced higher levels of WIF when compared with voluntary part-timers. This desire for more work hours is often driven by financial necessity, as caregivers who work fewer hours than they prefer may experience financial difficulties (Reynolds & Aletraris, 2007). Alternatively, the preference for more work hours may reflect the need for an activity that can counterbalance the stresses of caregiving and provide a break from that role (Hoff et al., 2014). Overall, our findings suggest associations between work mismatches and WIF, making it more challenging for individuals to fulfill their caregiving responsibilities.

Hypothesis 2 received partial support. Among the four types of flexible work arrangements, flexitime off was consistently and negatively associated with WIF, indicating that higher use of this arrangement was linked to lower levels of WIF for working caregivers. This finding aligns with the demands–resources approach to WIF (Voydanoff, 2005), where individuals use FWAs as resources to address the demands of work (Ray & Pana-Cryan, 2021). It is plausible that in our study, caregivers proactively planned and managed their caregiving responsibilities by utilizing flexitime off, leading to reduced WIF associated with balancing work and family roles.

Of the other FWA categories, the flexible schedule was positively related to WIF bivariate (against predictions) but was unrelated in multivariate analyses. Previous studies have also found inconsistent associations between FWAs and work interference with family (Allen et al., 2013), suggesting the need to examine the influence of other factors in this relationship. According to Lapierre and Allen (2012), one of the factors that contribute to WIF is the lack of control at work, which can make it challenging for individuals with low control to balance the demands of work and caregiving responsibilities. Although flexibility can serve as a resource, not everyone has the authority or autonomy to allocate resources in a way that can effectively prevent WIF (Lapierre & Allen, 2012). Therefore, using flexible work arrangements may put some individuals in precarious situations where they struggle to manage resource allocation choices efficiently, leading to increased conflict.

Hypothesis 3 was partially supported as only flexible time-off arrangements were found to moderate the relationship between involuntary part-timers and WIF.

Although there were no direct effects of involuntary part-time work status preference on WIF, the interaction effect indicates that time off plays a crucial role in influencing work–family conflict among involuntary part-time caregivers. For these caregivers, the ability to take time off when needed may act as a valuable resource. This resource may buffer against the challenges of balancing work and caregiving responsibilities, providing opportunities for caregivers to prioritize their family obligations without fear of losing their jobs or income. The demands-and-resources approach (Voydanoff, 2005), supports this interpretation, suggesting that resources like time-off flexibility can help alleviate work–family conflict. Additionally, this flexibility would be expected to benefit part-time workers more, as they are less likely to have the financial means to pay for formal care. Involuntary part-time work can be a source of financial strain and job insecurity (Kretsos & Livanos, 2016), and it may not be possible for workers to change their status due to factors outside of their control, such as the availability of work or their employer’s business needs. However, taking time off to attend to caregiving responsibilities can help alleviate some of the stress associated with trying to manage both work and family obligations simultaneously.

On the other hand, for involuntary part-time caregivers who do not have access to time-off arrangements, the absence of this resource may contribute to increased challenges in managing competing demands. The inability to utilize time-off flexibility means that these caregivers might face unexpected caregiving interruptions without the ability to plan or prepare for them. Care crises can occur at short notice and reflect the unpredictable and episodic nature of caregiving (Fine, 2012; Sims-Gould et al., 2008). Caregivers in precarious work may be reluctant to seek support from their employer, as doing so might impact their chances of job security. Employers have been shown to be less supportive when an individual’s caregiving circumstances are unpredictable (Breheny et al., 2022).

Moreover, utilizing time-off flexibility to attend to care needs may create tension upon return to work where co-workers are required to cover for the caregiver’s absence with the potential for initiating FIW and caregivers experiencing feelings of guilt (Fursman & Zodgekar, 2009). However, it is important to note that the present study did not directly examine the phenomenon of FIW. Although the results provide valuable insights into the relationship between work status preferences, time-off flexibility, and WIF, further investigation is needed to explore the potential implications of FIW in this context. Future studies could consider examining family interference with work as a complementary aspect to understand the reciprocal relationship between work and family roles among older adult caregivers.

The non-significant interaction effect between IVF work status preference and time off suggests that work–family balance involves a complex interplay of various factors. Although our findings suggest that time off may be beneficial for involuntary part-time caregivers, it might not have the same benefits for involuntary full-time caregivers. The greater work demands, and potential lack of control experienced by involuntary full-time caregivers, may make time off less effective in reducing WIF. Future research could investigate the specific challenges and needs of involuntary full-time caregivers and explore additional factors that could contribute to their work–family balance.

Further examination of the data reveals that more females experienced WIF compared with males. This finding is consistent with previous research showing that women often bear a higher burden of caregiving responsibilities, which can lead to increased WIF (Allen et al., 2013; Lapierre et al., 2017). Moreover, the significant effect of ELSI on WIF implies that economic factors associated with one’s life, such as financial stability and job security, may play a crucial role in determining the level of work–family conflict experienced by older adult caregivers. Those with higher ELSI scores may have greater financial resources and support, which could help them better manage work and caregiving responsibilities, potentially reducing WIF.

The nonsignificant interaction effects for flexible schedule, flexible place, and flexibility in number of hours worked suggest that these arrangements may not significantly address WIF among older adult caregivers. Although flexible work arrangements are often considered potential resources for work–life balance, their effectiveness in reducing WIF might vary for different individuals and situations. The study findings align with previous research reporting inconsistent associations between flexible work arrangements and WIF (Allen et al., 2013). It is crucial to recognize that work–family balance is multifaceted, influenced by various factors, including the nature of caregiving responsibilities, work demands, and organizational support.

To better support older adult caregivers, a comprehensive approach that considers multiple dimensions, including policies, resources, and individual needs, is essential. Tailored interventions and a supportive work culture can contribute to a more effective work–family balance for caregivers. Social policies must continue to pursue attempts to improve the fit between people and their jobs. For instance, designing comprehensive policies to combat precarious work will reduce social inequalities that may lead to mismatches between an individual’s preference and their present work. Further research can explore specific caregiver populations’ needs and challenges to guide targeted support measures.

### Limitations and Future Research

Several limitations should be noted. First, although the overall sample size was large, the number of involuntary part-timers was relatively small (*n* = 33) and findings, particularly interaction effects, should be interpreted with caution.

Second, the use of FWAs has been linked to a sense of control among employees. However, it is important to note that our study did not examine whether employees used FWAs voluntarily or involuntarily (Allen et al., 2013), such as during the COVID-19 pandemic when remote work was mandatory. Future research could address this limitation by investigating the relationship between perceived control and the voluntary nature of FWA use.

To gain a more comprehensive understanding of individual preferences for work arrangements, future studies could explore the perspectives of full-time employees who desire more work and part-time employees who desire less work. One approach suggested by Kalleberg (2008) is to have employers report the actual hours worked and preferred work hours or to ask participants directly about their preferred working hours. Additionally, the FWAs examined in our study were not specifically designed for caregiver situations, and thus may not have captured the types of arrangements that caregivers are more likely to need or use.

Third, previous research has highlighted the significance of employees perceiving that FWAs are accessible to them, even if they do not currently have a need to utilize them. This perception of access can serve as a psychological resource, providing individuals with a sense of control and support in managing their work and family responsibilities. Although our study examined utilization, the findings from past research on access shed light on the importance of creating a supportive work environment where employees are aware that FWAs are available if needed. The interplay between access and utilization warrants further exploration to better understand their joint influence on work–life balance outcomes. Future research should consider incorporating measures of both access and utilization to provide a more comprehensive understanding of the role of FWAs in promoting work–family integration among older adults with caregiving responsibilities.

## Conclusion

The results of our study suggest that when there are mismatches in work status preferences, it can lead to WIF and make it difficult for individuals to fulfill their familial responsibilities. However, our findings suggest that flexible work arrangements may not necessarily alleviate WIF among older adults who have both paid work and caregiving responsibilities. However, utilizing time-off flexibility to attend to family needs led to less WIF for individuals in involuntary part-time work.

As Putnam et al. (2014) suggest, to ensure that employees benefit from flexible arrangements, organizations should create an organizational climate that prevents employees from being subjected to criticisms from co-workers and negative job evaluations for using FWAs. Our present findings highlight the importance of employers and policymakers recognizing the limitations of current flexible work arrangements and design arrangements that align with the unique work circumstances of older workers. Overall, it is crucial to consider the impact of work status preferences and caregiving responsibilities on WIF among older workers. Creating a work environment that is supportive of the unique needs and circumstances of older workers can lead to a more engaged and productive workforce, while also enabling individuals to fulfill their familial responsibilities.

## Supplementary Material

igad115_suppl_Supplementary_Material
