# Supplementary material for: Reconciling Paid Work and Informal Caregiving Among Older Adults: Implications for Work Interference With Family
Source: Innov Aging. 2023 Oct 6;7(9):igad115. doi: 10.1093/geroni/igad115 (PMC10681359; doi:10.1093/geroni/igad115)
Supplement: igad115_suppl_Supplementary_Material [file igad115_suppl_supplementary_material.docx]

**Online Supplementary Material**

Flexible work arrangement survey (All items adapted from Bond, Thompson, Galinsky, & Prottas, 2002 and categorized into four distinct types based on the typology outlined in Pitt-Catsouphes et al. (2009)

Flexibility in the number of hours worked

1. Have input into the number of overtime hours you work
2. Have input to the numb of hours you work
3. Phased retirement
4. Structure job as a job share with another person where both receive their fair share of compensation and benefits

Time off

1. Take paid time off to volunteer in the community
2. Take sabbatical or career break. That is, take leave, paid or unpaid of one or more months and return to a comparable job
3. Take paid or unpaid time for education or training to improve job skills
4. Take extra unpaid vacation days
5. Take paid leave for caregiving or other personal or family responsibilities

Flexible place

1. Work part year, that is work for a reduce amount of time on an annual basis (e.g., work full time during autumn, winter and spring and take the summer off).
2. Reduce your work hours and work on a part time basis while remaining the same position or at the same level

Flexible Schedule

1. Make choices about which shift you work
2. Choose a work schedule that varies from the typical schedule at your worksite
3. Compressed workweek by working longer hours on fewer days for at least part of the year
4. Occasional request changes in starting and quitting times
5. Frequent changes in starting and quitting time
